# Dibromidobis(*N*,*N*-diethyl­dithio­carbamato-κ^2^ *S*,*S*′)tetra-μ_3_-sulfido-dicopper(I)dimolybdenum(V) isopropanol disolvate

**DOI:** 10.1107/S1600536808035241

**Published:** 2008-11-13

**Authors:** Qing Zhang, Min Liu

**Affiliations:** a College of Chemistry and Chemical Engineering, Jiangxi Science and Technology Normal University, Nanchang 330013, People’s Republic of China

## Abstract

The mol­ecule of the title compound, [Cu_2_Mo_2_Br_2_(C_7_H_14_NS_2_)_2_S_4_]·2C_3_H_7_OH, comprises one [(*i*-C_3_H_7_)_2_NCS_2_]_2_Mo_2_S_4_ unit and two CuBr units held together by six Cu—μ_3_-S bonds, thus forming a cubane-like Mo_2_S_4_Cu_2_ core. Intramolecular O—H⋯S hydrogen bonds may stabilize the structure. Two methyl groups of the two independent solvent molecules are disordered over two positions and were refined with occupancies of 0.733 (12) and 0.267 (12).

## Related literature

For sulfido-bridged dinuclear complexes with an *M*_2_S_4_ core (*M* = Mo, W), see: Hidai *et al.* (1999 ▶); Lang *et al.* (2003 ▶); Curtis *et al.* (1997 ▶); Stiefel *et al.* (1985 ▶); Wu *et al.* (1990 ▶).
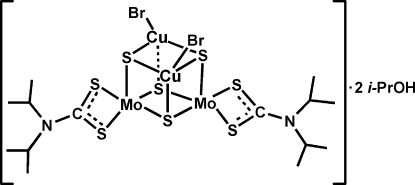


## Experimental

### 

#### Crystal data

[Cu_2_Mo_2_Br_2_(C_7_H_14_NS_2_)_2_S_4_]·2C_3_H_8_O
*M* *_r_* = 1079.91
Triclinic, 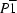

*a* = 12.515 (3) Å
*b* = 12.734 (3) Å
*c* = 12.759 (3) Å
α = 107.76 (3)°
β = 108.26 (3)°
γ = 90.12 (3)°
*V* = 1828.2 (9) Å^3^
*Z* = 2
Mo *K*α radiation
μ = 4.48 mm^−1^
*T* = 291 (2) K
0.30 × 0.29 × 0.20 mm

#### Data collection

Rigaku Mercury diffractometer
Absorption correction: multi-scan (Jacobson, 1998 ▶) *T*_min_ = 0.284, *T*_max_ = 0.408
17918 measured reflections
6703 independent reflections
5814 reflections with *I* > 2σ(*I*)
*R*_int_ = 0.032

#### Refinement

*R*[*F* ^2^ > 2σ(*F* ^2^)] = 0.055
*wR*(*F* ^2^) = 0.121
*S* = 1.12
6703 reflections
353 parameters
10 restraints
H-atom parameters constrained
Δρ_max_ = 1.90 e Å^−3^
Δρ_min_ = −2.13 e Å^−3^

### 

Data collection: *CrystalClear* (Rigaku/MSC, 2001 ▶); cell refinement: *CrystalClear*; data reduction: *CrystalStructure* (Rigaku/MSC, 2004 ▶); program(s) used to solve structure: *SHELXS97* (Sheldrick, 2008 ▶); program(s) used to refine structure: *SHELXL97* (Sheldrick, 2008 ▶); molecular graphics: *SHELXTL* (Sheldrick, 2008 ▶); software used to prepare material for publication: *SHELXTL*.

## Supplementary Material

Crystal structure: contains datablocks I, global. DOI: 10.1107/S1600536808035241/hk2539sup1.cif

Structure factors: contains datablocks I. DOI: 10.1107/S1600536808035241/hk2539Isup2.hkl

Additional supplementary materials:  crystallographic information; 3D view; checkCIF report

## Figures and Tables

**Table 1:** Hydrogen-bond geometry (Å, °)

*D*—H⋯*A*	*D*—H	H⋯*A*	*D*⋯*A*	*D*—H⋯*A*
O2—H2*D*⋯S5	0.82	2.47	3.199 (8)	149
O2—H2*D*⋯S6	0.82	2.59	3.258 (8)	139

**Fig. 1.**
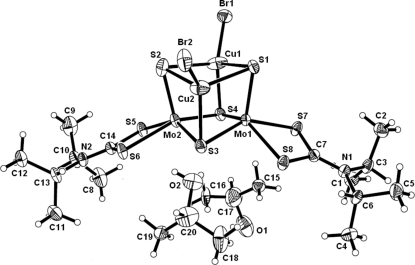
The molecular structure of the title molecule, with the atom-numbering scheme. Displacement ellipsoids are drawn at the 40% probability level.

## supplementary crystallographic information

### Comment

In the past decades, chemistry of the sulfido-bridged dinuclear complexes with an M_2_S_4_ core (M = Mo, W) and various transition metals has been intensively investigated. For example, precursors [(dtc)_2_Mo_2_S_2_(µ-S)_2_] (dtc = S_2_CNEt_2_) (Hidai *et al.*, 1999; Lang *et al.*, 2003) and [Cp^x^_2_Mo_2_S_2_(µ-S)_2_] (Cp^x^ = pentamethyl-, pentaethyl- or pentabutyl-cyclopentadienyl) (Curtis *et al.*, 1997; Stiefel *et al.*, 1985) and [Et_4_N]_2_[(edt)_2_Mo_2_S_2_(µ-S)_2_] (edt = ethanedithiolate) (Wu *et al.*, 1990) were shown to react with transition metals to form both incomplete [Mo_2_MS_4_] and complete [Mo_2_M_2_S_4_] cubane-like clusters. We report herein the formation of a complete cubane-like [Mo_2_Cu_2_S_4_] by using [(i-C_3_H_7_)_2_NCS_2_]_2_Mo_2_S_4_ as the starting material to react with two equivalents of CuBr.

The title molecule contains one [(i-C_3_H_7_)_2_NCS_2_]_2_Mo_2_S_4_ moiety and two CuBr units, which are assembled into a distorted Mo_2_Cu_2_S_4_ cubane-like core (Fig. 1). The formal oxidation states for each Mo and Cu remain + 5 and + 1, respectively. Each Mo center is coordinated by three µ_3_-S atoms, and the two S atoms of an [(i-C_3_H_7_)_2_NCS_2_] group, forming a distorted square pyramidal geometry, while each Cu atom is tetrahedrally coordinated by three µ_3_-S atoms and a terminal bromide. The Mo-S bonds are in the range of 2.1621 (19)-2.4465 (18) Å, due to the different S atoms coordinated. The Cu-S(terminal) bonds [average value: 2.436 (2) Å] are longer than the other Cu-S bonds [average value: 2.211 (2) Å]. The Mo···Mo [2.7874 (10) Å] and Mo···Cu [average value: 2.8114 (15) Å] distances and the Cu-Br bonds [average value: 2.2812 (15) Å] have normal values. Intramolecular O-H···S hydrogen bonds (Table 1) may be effective in the stabilization of the structure.

### Experimental

For the preparation of the title compound, [(i-C_3_H_7_)_2_NCS_2_]_2_Mo_2_S_4_ (0.49 g, 0.5 mmol), and CuBr (0.144 g, 1.0 mmol) were added into CH_2_Cl_2_ solution (20 ml). The mixture was stirred at room temperature for 0.5 h, and the dark-red suspension gradually turned into dark red solution, and then filtered. The filtrate was layered with isopropyl alcohol (30 ml) to produce dark red crystals in 4 d.

### Refinement

The C15, C16 and C19 methyl groups in di-isopropyl alcohol solvate were disordered over two positions. During the refinement process the disordered atoms were refined with occupancies of 0.733 (12) for C15, H15A, H15B, H15C, C16, H16A, H16B, H16C, C19, H19A, H19B, H19C and 0.267 (12) for C15A, H15D, H15E, H15F, C16A, H16D, H16E, H16F, C19A, H19D, H19E, H19F, respectively. The C15 and C15A atoms were refined isotropically. H atoms were positioned geometrically, with O-H = 0.82 Å (for OH) and C-H = 0.98 and 0.96 Å for methine and methyl H, respectively, and constrained to ride on their parent atoms with U_iso_(H) = xU_eq_(C,O), where x = 1.2 for methine H and x = 1.5 for all other H atoms.

### Crystal data

**Table:**

[Cu_2_Mo_2_Br_2_(C_7_H_14_NS_2_)_2_S_4_]·2C_3_H_8_O	*Z* = 2
*M**_r_* = 1079.91	*F*_000_ = 1068
Triclinic, *P*1	*D*_x_ = 1.962 Mg m^−^^3^
Hall symbol: -P 1	Mo *K*α radiation λ = 0.71073 Å
*a* = 12.515 (3) Å	Cell parameters from 6125 reflections
*b* = 12.734 (3) Å	θ = 3.3–25.4º
*c* = 12.759 (3) Å	µ = 4.48 mm^−^^1^
α = 107.76 (3)º	*T* = 291 (2) K
β = 108.26 (3)º	Block, dark red
γ = 90.12 (3)º	0.30 × 0.29 × 0.20 mm
*V* = 1828.2 (9) Å^3^	

### Data collection

**Table:**

Rigaku Mercury diffractometer	6703 independent reflections
Radiation source: fine-focus sealed tube	5814 reflections with *I* > 2σ(*I*)
Monochromator: graphite	*R*_int_ = 0.032
*T* = 291(2) K	θ_max_ = 25.4º
ω scans	θ_min_ = 3.3º
Absorption correction: multi-scan(Jacobson, 1998)	*h* = −15→15
*T*_min_ = 0.284, *T*_max_ = 0.408	*k* = −15→15
17918 measured reflections	*l* = −15→15

### Refinement

**Table:**

Refinement on *F*^2^	Secondary atom site location: difference Fourier map
Least-squares matrix: full	Hydrogen site location: inferred from neighbouring sites
*R*[*F*^2^ > 2σ(*F*^2^)] = 0.055	H-atom parameters constrained
*wR*(*F*^2^) = 0.121	*w* = 1/[σ^2^(*F*_o_^2^) + (0.0376*P*)^2^ + 11.7921*P*] where *P* = (*F*_o_^2^ + 2*F*_c_^2^)/3
*S* = 1.12	(Δ/σ)_max_ = 0.006
6703 reflections	Δρ_max_ = 1.90 e Å^−^^3^
353 parameters	Δρ_min_ = −2.13 e Å^−^^3^
10 restraints	Extinction correction: none
Primary atom site location: structure-invariant direct methods	

### Special details

**Table:**

Geometry. All esds (except the esd in the dihedral angle between two l.s. planes) are estimated using the full covariance matrix. The cell esds are taken into account individually in the estimation of esds in distances, angles and torsion angles; correlations between esds in cell parameters are only used when they are defined by crystal symmetry. An approximate (isotropic) treatment of cell esds is used for estimating esds involving l.s. planes.
Refinement. Refinement of F^2^ against ALL reflections. The weighted R-factor wR and goodness of fit S are based on F^2^, conventional R-factors R are based on F, with F set to zero for negative F^2^. The threshold expression of F^2^ > 2sigma(F^2^) is used only for calculating R-factors(gt) etc. and is not relevant to the choice of reflections for refinement. R-factors based on F^2^ are statistically about twice as large as those based on F, and R- factors based on ALL data will be even larger.

### Fractional atomic coordinates and isotropic or equivalent isotropic displacement parameters (Å^2^)

**Table:**

	*x*	*y*	*z*	*U*_iso_*/*U*_eq_	Occ. (<1)
Mo1	1.00881 (5)	0.18736 (4)	0.39883 (5)	0.02348 (15)	
Mo2	1.16712 (5)	0.20599 (5)	0.29120 (5)	0.02768 (16)	
Cu1	1.12203 (10)	0.00501 (8)	0.33253 (10)	0.0544 (3)	
Cu2	1.23373 (9)	0.24097 (8)	0.53604 (10)	0.0517 (3)	
Br1	1.13513 (7)	−0.18080 (6)	0.29790 (7)	0.0441 (2)	
Br2	1.37543 (7)	0.30267 (8)	0.71075 (7)	0.0535 (3)	
S1	1.09552 (15)	0.10190 (15)	0.51854 (15)	0.0308 (4)	
S2	1.29693 (17)	0.12006 (18)	0.3783 (2)	0.0473 (5)	
S3	1.13192 (15)	0.34620 (14)	0.44228 (15)	0.0301 (4)	
S4	1.00310 (14)	0.07858 (14)	0.21230 (14)	0.0281 (4)	
S5	1.17527 (16)	0.15164 (14)	0.09288 (15)	0.0323 (4)	
S6	1.28036 (15)	0.35219 (14)	0.27486 (15)	0.0311 (4)	
S7	0.81395 (14)	0.10577 (14)	0.33380 (16)	0.0327 (4)	
S8	0.90078 (15)	0.32398 (14)	0.48497 (16)	0.0325 (4)	
O1	0.6199 (7)	0.1776 (6)	−0.1619 (6)	0.087 (2)	
H1D	0.6271	0.1595	−0.2265	0.131*	
O2	1.0055 (6)	0.3083 (5)	0.1913 (6)	0.0697 (19)	
H2D	1.0646	0.2930	0.1781	0.104*	
N1	0.6852 (5)	0.2439 (4)	0.4372 (5)	0.0284 (12)	
N2	1.3096 (5)	0.3023 (5)	0.0653 (5)	0.0293 (13)	
C1	0.5412 (6)	0.1342 (7)	0.2459 (6)	0.0413 (18)	
H1A	0.5335	0.2023	0.2279	0.062*	
H1B	0.4691	0.0901	0.2127	0.062*	
H1C	0.5947	0.0940	0.2140	0.062*	
C2	0.5971 (7)	0.0577 (6)	0.4167 (8)	0.046 (2)	
H2A	0.6226	0.0806	0.5003	0.069*	
H2B	0.6520	0.0160	0.3886	0.069*	
H2C	0.5260	0.0122	0.3862	0.069*	
C3	0.5824 (6)	0.1597 (6)	0.3769 (7)	0.0349 (16)	
H3A	0.5220	0.1956	0.4033	0.042*	
C4	0.6128 (7)	0.4224 (6)	0.4354 (7)	0.0429 (19)	
H4A	0.6557	0.4262	0.3859	0.064*	
H4B	0.6087	0.4959	0.4831	0.064*	
H4C	0.5377	0.3876	0.3883	0.064*	
C5	0.6097 (8)	0.3443 (7)	0.5956 (8)	0.050 (2)	
H5A	0.6503	0.3014	0.6423	0.075*	
H5B	0.5345	0.3081	0.5516	0.075*	
H5C	0.6056	0.4169	0.6454	0.075*	
C6	0.6703 (6)	0.3545 (6)	0.5132 (6)	0.0298 (15)	
H6A	0.7460	0.3928	0.5609	0.036*	
C7	0.7831 (6)	0.2252 (5)	0.4229 (6)	0.0287 (15)	
C8	1.1642 (8)	0.2257 (9)	−0.1322 (7)	0.063 (3)	
H8A	1.1360	0.2969	−0.1193	0.094*	
H8B	1.1222	0.1780	−0.1100	0.094*	
H8C	1.1559	0.1935	−0.2132	0.094*	
C9	1.3407 (9)	0.1315 (7)	−0.0753 (8)	0.061 (3)	
H9A	1.4192	0.1456	−0.0281	0.091*	
H9B	1.3342	0.0982	−0.1557	0.091*	
H9C	1.3022	0.0820	−0.0518	0.091*	
C10	1.2881 (7)	0.2392 (6)	−0.0598 (6)	0.0401 (19)	
H10A	1.3274	0.2847	−0.0889	0.048*	
C11	1.3276 (8)	0.4937 (7)	0.0623 (9)	0.056 (2)	
H11A	1.2572	0.5032	0.0775	0.084*	
H11B	1.3132	0.4691	−0.0203	0.084*	
H11C	1.3750	0.5630	0.0975	0.084*	
C12	1.4996 (7)	0.3893 (7)	0.0935 (7)	0.046 (2)	
H12A	1.5341	0.3352	0.1281	0.069*	
H12B	1.5484	0.4578	0.1287	0.069*	
H12C	1.4879	0.3629	0.0113	0.069*	
C13	1.3864 (6)	0.4082 (6)	0.1130 (6)	0.0337 (16)	
H13A	1.4011	0.4361	0.1974	0.040*	
C14	1.2634 (6)	0.2720 (5)	0.1317 (6)	0.0281 (15)	
C15	0.7206 (16)	0.1461 (12)	0.0386 (12)	0.041 (3)*	0.733 (12)
H15A	0.7706	0.0973	0.0688	0.061*	0.733 (12)
H15B	0.6436	0.1170	0.0193	0.061*	0.733 (12)
H15C	0.7353	0.2182	0.0964	0.061*	0.733 (12)
C15A	0.722 (4)	0.186 (5)	0.057 (3)	0.041 (3)*	0.267 (12)
H15D	0.7931	0.2152	0.1172	0.061*	0.267 (12)
H15E	0.6914	0.1219	0.0661	0.061*	0.267 (12)
H15F	0.6700	0.2418	0.0613	0.061*	0.267 (12)
C16	0.8461 (8)	0.2073 (9)	−0.0314 (8)	0.039 (3)	0.733 (12)
H16A	0.8528	0.2791	0.0251	0.058*	0.733 (12)
H16B	0.8590	0.2158	−0.0989	0.058*	0.733 (12)
H16C	0.9012	0.1639	0.0017	0.058*	0.733 (12)
C16A	0.750 (3)	0.181 (5)	0.0666 (18)	0.039 (3)	0.267 (12)
H16D	0.8166	0.1527	0.1055	0.058*	0.267 (12)
H16E	0.6846	0.1478	0.0724	0.058*	0.267 (12)
H16F	0.7567	0.2603	0.1025	0.058*	0.267 (12)
C17	0.7390 (11)	0.1546 (12)	−0.0634 (10)	0.092 (4)	
H17	0.7484	0.0778	−0.1029	0.111*	
C18	0.8873 (8)	0.4760 (11)	0.2435 (8)	0.080 (4)	
H18A	0.8899	0.5548	0.2593	0.120*	
H18B	0.8831	0.4585	0.3103	0.120*	
H18C	0.8218	0.4401	0.1775	0.120*	
C19	1.0116 (11)	0.4568 (14)	0.1142 (12)	0.059 (4)	0.733 (12)
H19A	1.0792	0.4282	0.1019	0.088*	0.733 (12)
H19B	1.0174	0.5354	0.1280	0.088*	0.733 (12)
H19C	0.9474	0.4214	0.0464	0.088*	0.733 (12)
C19A	0.991 (3)	0.388 (4)	0.100 (4)	0.059 (4)	0.267 (12)
H19D	0.9542	0.4360	0.0569	0.088*	0.267 (12)
H19E	0.9475	0.3167	0.0671	0.088*	0.267 (12)
H19F	1.0654	0.3808	0.0950	0.088*	0.267 (12)
C20	0.9974 (10)	0.4343 (10)	0.2164 (10)	0.083 (3)	
H20	1.0628	0.4732	0.2846	0.099*	

### Atomic displacement parameters (Å^2^)

**Table:**

	*U*^11^	*U*^22^	*U*^33^	*U*^12^	*U*^13^	*U*^23^
Mo1	0.0218 (3)	0.0244 (3)	0.0245 (3)	−0.0001 (2)	0.0092 (2)	0.0066 (2)
Mo2	0.0320 (3)	0.0283 (3)	0.0232 (3)	−0.0066 (2)	0.0126 (2)	0.0052 (2)
Cu1	0.0570 (7)	0.0372 (6)	0.0485 (6)	0.0155 (5)	−0.0029 (5)	0.0061 (5)
Cu2	0.0422 (6)	0.0401 (6)	0.0523 (6)	−0.0043 (4)	−0.0116 (5)	0.0145 (5)
Br1	0.0499 (5)	0.0361 (4)	0.0474 (5)	0.0070 (4)	0.0162 (4)	0.0146 (4)
Br2	0.0384 (5)	0.0843 (7)	0.0297 (4)	−0.0176 (4)	0.0042 (3)	0.0146 (4)
S1	0.0307 (9)	0.0365 (9)	0.0284 (9)	−0.0022 (7)	0.0110 (7)	0.0139 (8)
S2	0.0355 (11)	0.0513 (12)	0.0539 (13)	−0.0013 (9)	0.0220 (10)	0.0076 (10)
S3	0.0314 (9)	0.0260 (9)	0.0325 (9)	−0.0032 (7)	0.0133 (8)	0.0061 (7)
S4	0.0302 (9)	0.0285 (9)	0.0240 (8)	−0.0036 (7)	0.0095 (7)	0.0057 (7)
S5	0.0387 (10)	0.0319 (9)	0.0249 (9)	−0.0102 (7)	0.0161 (8)	0.0017 (7)
S6	0.0368 (10)	0.0311 (9)	0.0244 (8)	−0.0107 (7)	0.0157 (7)	0.0019 (7)
S7	0.0233 (9)	0.0280 (9)	0.0390 (10)	−0.0016 (7)	0.0123 (8)	−0.0022 (8)
S8	0.0265 (9)	0.0267 (9)	0.0405 (10)	−0.0028 (7)	0.0152 (8)	0.0015 (8)
O1	0.117 (7)	0.081 (5)	0.064 (5)	−0.019 (5)	0.042 (5)	0.010 (4)
O2	0.081 (5)	0.060 (4)	0.067 (4)	0.012 (4)	0.020 (4)	0.024 (4)
N1	0.026 (3)	0.026 (3)	0.029 (3)	−0.002 (2)	0.008 (2)	0.003 (2)
N2	0.033 (3)	0.033 (3)	0.023 (3)	−0.002 (2)	0.016 (2)	0.004 (2)
C1	0.033 (4)	0.042 (4)	0.039 (4)	0.000 (3)	0.004 (3)	0.007 (4)
C2	0.039 (5)	0.041 (4)	0.057 (5)	−0.007 (4)	0.011 (4)	0.021 (4)
C3	0.022 (4)	0.035 (4)	0.043 (4)	0.000 (3)	0.012 (3)	0.004 (3)
C4	0.046 (5)	0.037 (4)	0.044 (5)	0.011 (4)	0.014 (4)	0.012 (4)
C5	0.060 (6)	0.044 (5)	0.057 (5)	0.005 (4)	0.037 (5)	0.011 (4)
C6	0.027 (4)	0.032 (4)	0.028 (4)	0.004 (3)	0.012 (3)	0.001 (3)
C7	0.033 (4)	0.026 (3)	0.028 (4)	0.003 (3)	0.014 (3)	0.006 (3)
C8	0.057 (6)	0.090 (7)	0.031 (5)	−0.026 (5)	−0.002 (4)	0.022 (5)
C9	0.077 (7)	0.053 (5)	0.049 (5)	−0.005 (5)	0.041 (5)	−0.010 (4)
C10	0.053 (5)	0.045 (4)	0.020 (4)	−0.012 (4)	0.016 (3)	0.002 (3)
C11	0.064 (6)	0.043 (5)	0.074 (6)	0.002 (4)	0.032 (5)	0.026 (5)
C12	0.045 (5)	0.044 (5)	0.051 (5)	−0.007 (4)	0.022 (4)	0.014 (4)
C13	0.040 (4)	0.032 (4)	0.031 (4)	−0.012 (3)	0.016 (3)	0.008 (3)
C14	0.029 (4)	0.028 (4)	0.027 (4)	−0.002 (3)	0.010 (3)	0.007 (3)
C16	0.020 (5)	0.068 (7)	0.018 (4)	−0.017 (5)	0.002 (4)	0.005 (4)
C16A	0.020 (5)	0.068 (7)	0.018 (4)	−0.017 (5)	0.002 (4)	0.005 (4)
C17	0.089 (9)	0.125 (11)	0.087 (9)	0.027 (8)	0.045 (7)	0.051 (8)
C18	0.056 (6)	0.154 (11)	0.031 (5)	−0.012 (7)	0.010 (4)	0.034 (6)
C19	0.047 (7)	0.092 (12)	0.072 (8)	0.031 (8)	0.028 (6)	0.065 (10)
C19A	0.047 (7)	0.092 (12)	0.072 (8)	0.031 (8)	0.028 (6)	0.065 (10)
C20	0.093 (9)	0.084 (8)	0.054 (6)	0.015 (7)	0.010 (6)	0.011 (6)

### Geometric parameters (Å, °)

**Table:**

Mo1—Mo2	2.7874 (10)	C8—H8B	0.9600
Mo1—Cu1	2.7715 (14)	C8—H8C	0.9600
Mo1—Cu2	2.7618 (16)	C9—C10	1.508 (12)
Mo1—S1	2.1621 (19)	C9—H9A	0.9600
Mo1—S3	2.3535 (19)	C9—H9B	0.9600
Mo1—S4	2.3386 (19)	C9—H9C	0.9600
Mo1—S7	2.4310 (19)	C10—N2	1.486 (8)
Mo1—S8	2.4160 (19)	C10—H10A	0.9800
Mo2—Cu1	2.8547 (14)	C11—C13	1.510 (11)
Mo2—Cu2	2.8582 (15)	C11—H11A	0.9600
Mo2—S2	2.166 (2)	C11—H11B	0.9600
Mo2—S3	2.352 (2)	C11—H11C	0.9600
Mo2—S4	2.356 (2)	C12—C13	1.522 (10)
Mo2—S5	2.4465 (18)	C12—H12A	0.9600
Mo2—S6	2.4349 (18)	C12—H12B	0.9600
Cu1—Br1	2.2867 (14)	C12—H12C	0.9600
Cu1—S1	2.444 (2)	C13—N2	1.497 (8)
Cu1—S2	2.437 (2)	C13—H13A	0.9800
Cu1—S4	2.213 (2)	C14—N2	1.306 (8)
Cu2—Br2	2.2756 (16)	C15—H15A	0.9600
Cu2—S1	2.383 (2)	C15—H15B	0.9600
Cu2—S2	2.481 (3)	C15—H15C	0.9600
Cu2—S3	2.209 (2)	C15A—H15D	0.9600
S5—C14	1.731 (7)	C15A—H15E	0.9600
S6—C14	1.744 (7)	C15A—H15F	0.9600
S7—C7	1.726 (7)	C16—H16A	0.9600
S8—C7	1.741 (7)	C16—H16B	0.9600
O1—H1D	0.8200	C16—H16C	0.9600
O2—H2D	0.8200	C16A—H16D	0.9600
C1—C3	1.515 (10)	C16A—H16E	0.9600
C1—H1A	0.9600	C16A—H16F	0.9600
C1—H1B	0.9600	C17—C16	1.378 (15)
C1—H1C	0.9600	C17—C15	1.425 (17)
C2—C3	1.525 (10)	C17—C15A	1.544 (19)
C2—H2A	0.9600	C17—C16A	1.547 (18)
C2—H2B	0.9600	C17—O1	1.715 (15)
C2—H2C	0.9600	C17—H17	0.9800
C3—N1	1.505 (8)	C18—H18A	0.9600
C3—H3A	0.9800	C18—H18B	0.9600
C4—C6	1.526 (10)	C18—H18C	0.9600
C4—H4A	0.9600	C19—H19A	0.9600
C4—H4B	0.9600	C19—H19B	0.9600
C4—H4C	0.9600	C19—H19C	0.9600
C5—C6	1.510 (10)	C19A—H19D	0.9600
C5—H5A	0.9600	C19A—H19E	0.9600
C5—H5B	0.9600	C19A—H19F	0.9600
C5—H5C	0.9600	C20—C19A	1.40 (4)
C6—N1	1.497 (8)	C20—C19	1.479 (17)
C6—H6A	0.9800	C20—O2	1.546 (13)
C7—N1	1.306 (8)	C20—C18	1.579 (12)
C8—C10	1.513 (12)	C20—H20	0.9800
C8—H8A	0.9600		
			
S1—Mo1—S4	107.30 (7)	C10—C8—H8B	109.5
S1—Mo1—S3	105.60 (7)	H8A—C8—H8B	109.5
S4—Mo1—S3	104.32 (7)	C10—C8—H8C	109.5
S1—Mo1—S8	110.87 (7)	H8A—C8—H8C	109.5
S4—Mo1—S8	137.84 (7)	H8B—C8—H8C	109.5
S3—Mo1—S8	82.15 (6)	C6—C5—H5A	109.5
S1—Mo1—S7	102.40 (7)	C6—C5—H5B	109.5
S4—Mo1—S7	83.56 (7)	H5A—C5—H5B	109.5
S3—Mo1—S7	146.89 (7)	C6—C5—H5C	109.5
S8—Mo1—S7	71.52 (6)	H5A—C5—H5C	109.5
S1—Mo1—Cu2	56.33 (6)	H5B—C5—H5C	109.5
S4—Mo1—Cu2	107.39 (6)	C3—C2—H2A	109.5
S3—Mo1—Cu2	50.40 (6)	C3—C2—H2B	109.5
S8—Mo1—Cu2	108.24 (6)	H2A—C2—H2B	109.5
S7—Mo1—Cu2	157.85 (6)	C3—C2—H2C	109.5
S1—Mo1—Cu1	57.82 (6)	H2A—C2—H2C	109.5
S4—Mo1—Cu1	50.46 (5)	H2B—C2—H2C	109.5
S3—Mo1—Cu1	106.97 (6)	C3—C1—H1A	109.5
S8—Mo1—Cu1	166.67 (6)	C3—C1—H1B	109.5
S7—Mo1—Cu1	102.73 (6)	H1A—C1—H1B	109.5
Cu2—Mo1—Cu1	72.36 (5)	C3—C1—H1C	109.5
S1—Mo1—Mo2	101.97 (5)	H1A—C1—H1C	109.5
S4—Mo1—Mo2	53.87 (5)	H1B—C1—H1C	109.5
S3—Mo1—Mo2	53.66 (5)	C6—C4—H4A	109.5
S8—Mo1—Mo2	130.76 (5)	C6—C4—H4B	109.5
S7—Mo1—Mo2	135.61 (5)	H4A—C4—H4B	109.5
Cu2—Mo1—Mo2	62.00 (4)	C6—C4—H4C	109.5
Cu1—Mo1—Mo2	61.80 (4)	H4A—C4—H4C	109.5
S2—Mo2—S3	105.05 (8)	H4B—C4—H4C	109.5
S2—Mo2—S4	104.02 (8)	N2—C13—C11	109.8 (6)
S3—Mo2—S4	103.81 (7)	N2—C13—C12	111.4 (6)
S2—Mo2—S6	101.58 (8)	C11—C13—C12	112.6 (6)
S3—Mo2—S6	85.72 (6)	N2—C13—H13A	107.6
S4—Mo2—S6	149.13 (7)	C11—C13—H13A	107.6
S2—Mo2—S5	103.59 (8)	C12—C13—H13A	107.6
S3—Mo2—S5	146.24 (7)	N1—C7—S7	126.4 (5)
S4—Mo2—S5	86.05 (7)	N1—C7—S8	123.9 (5)
S6—Mo2—S5	71.31 (7)	S7—C7—S8	109.6 (4)
S2—Mo2—Mo1	98.63 (6)	N2—C10—C9	111.9 (6)
S3—Mo2—Mo1	53.70 (5)	N2—C10—C8	112.6 (7)
S4—Mo2—Mo1	53.29 (5)	C9—C10—C8	113.5 (7)
S6—Mo2—Mo1	138.23 (5)	N2—C10—H10A	106.1
S5—Mo2—Mo1	137.45 (5)	C9—C10—H10A	106.1
S2—Mo2—Cu1	56.09 (6)	C8—C10—H10A	106.1
S3—Mo2—Cu1	104.42 (5)	N1—C6—C5	112.3 (6)
S4—Mo2—Cu1	49.13 (5)	N1—C6—C4	108.4 (6)
S6—Mo2—Cu1	156.98 (6)	C5—C6—C4	113.6 (6)
S5—Mo2—Cu1	106.21 (6)	N1—C6—H6A	107.4
Mo1—Mo2—Cu1	58.83 (4)	C5—C6—H6A	107.4
S2—Mo2—Cu2	57.22 (7)	C4—C6—H6A	107.4
S3—Mo2—Cu2	49.00 (5)	N1—C3—C1	112.6 (6)
S4—Mo2—Cu2	103.90 (6)	N1—C3—C2	112.3 (6)
S6—Mo2—Cu2	104.35 (6)	C1—C3—C2	114.5 (6)
S5—Mo2—Cu2	159.79 (6)	N1—C3—H3A	105.5
Mo1—Mo2—Cu2	58.56 (4)	C1—C3—H3A	105.5
Cu1—Mo2—Cu2	69.75 (5)	C2—C3—H3A	105.5
S4—Cu1—Br1	124.95 (7)	N2—C14—S5	126.6 (5)
S4—Cu1—S2	100.02 (9)	N2—C14—S6	123.5 (5)
Br1—Cu1—S2	116.22 (8)	S5—C14—S6	109.9 (4)
S4—Cu1—S1	102.17 (8)	C16—C17—C15	108.4 (12)
Br1—Cu1—S1	114.44 (7)	C16—C17—C15A	100 (2)
S2—Cu1—S1	93.70 (8)	C16—C17—C16A	90.1 (16)
S4—Cu1—Mo1	54.58 (5)	C16—C17—O1	125.5 (10)
Br1—Cu1—Mo1	148.85 (6)	C15—C17—O1	113.2 (11)
S2—Cu1—Mo1	92.75 (7)	C15A—C17—O1	108.3 (18)
S1—Cu1—Mo1	48.48 (5)	C16A—C17—O1	121 (2)
S4—Cu1—Mo2	53.62 (6)	C16—C17—H17	102.0
Br1—Cu1—Mo2	150.08 (6)	C15—C17—H17	102.0
S2—Cu1—Mo2	47.51 (6)	C15A—C17—H17	120.4
S1—Cu1—Mo2	93.37 (6)	C16A—C17—H17	115.2
Mo1—Cu1—Mo2	59.37 (3)	O1—C17—H17	102.0
S3—Cu2—Br2	125.87 (7)	C19A—C20—O2	72.2 (18)
S3—Cu2—S1	103.11 (8)	C19—C20—O2	104.5 (10)
Br2—Cu2—S1	115.75 (7)	C19A—C20—C18	118.1 (18)
S3—Cu2—S2	99.62 (9)	C19—C20—C18	113.8 (9)
Br2—Cu2—S2	112.85 (8)	O2—C20—C18	115.1 (10)
S1—Cu2—S2	94.10 (8)	C19A—C20—H20	129.3
S3—Cu2—Mo1	55.17 (5)	C19—C20—H20	107.7
Br2—Cu2—Mo1	153.00 (6)	O2—C20—H20	107.7
S1—Cu2—Mo1	49.02 (5)	C18—C20—H20	107.7
S2—Cu2—Mo1	92.04 (7)	C20—C18—H18A	109.5
S3—Cu2—Mo2	53.47 (6)	C20—C18—H18B	109.5
Br2—Cu2—Mo2	146.41 (6)	H18A—C18—H18B	109.5
S1—Cu2—Mo2	94.60 (6)	C20—C18—H18C	109.5
S2—Cu2—Mo2	47.21 (6)	H18A—C18—H18C	109.5
Mo1—Cu2—Mo2	59.44 (4)	H18B—C18—H18C	109.5
Cu1—S4—Mo1	74.96 (6)	C7—N1—C6	120.0 (5)
Cu1—S4—Mo2	77.26 (7)	C7—N1—C3	123.1 (6)
Mo1—S4—Mo2	72.84 (6)	C6—N1—C3	116.9 (5)
C14—S6—Mo2	89.4 (2)	C14—N2—C10	124.0 (6)
C7—S7—Mo1	89.2 (2)	C14—N2—C13	120.0 (5)
C7—S8—Mo1	89.3 (2)	C10—N2—C13	116.0 (5)
Mo2—S2—Cu1	76.40 (7)	C17—O1—H1D	109.5
Mo2—S2—Cu2	75.57 (7)	C20—O2—H2D	109.5
Cu1—S2—Cu2	83.22 (8)	C17—C15—H15A	109.5
Mo1—S1—Cu2	74.65 (6)	C17—C15—H15B	109.5
Mo1—S1—Cu1	73.70 (6)	C17—C15—H15C	109.5
Cu2—S1—Cu1	85.16 (7)	C20—C19—H19A	109.5
C14—S5—Mo2	89.3 (2)	C20—C19—H19B	109.5
Cu2—S3—Mo2	77.53 (7)	H19A—C19—H19B	109.5
Cu2—S3—Mo1	74.43 (6)	C20—C19—H19C	109.5
Mo2—S3—Mo1	72.65 (6)	H19A—C19—H19C	109.5
C10—C9—H9A	109.5	H19B—C19—H19C	109.5
C10—C9—H9B	109.5	C17—C16—H16A	109.5
H9A—C9—H9B	109.5	C17—C16—H16B	109.5
C10—C9—H9C	109.5	C17—C16—H16C	109.5
H9A—C9—H9C	109.5	C20—C19A—H19D	109.5
H9B—C9—H9C	109.5	C20—C19A—H19E	109.5
C13—C12—H12A	109.5	H19D—C19A—H19E	109.5
C13—C12—H12B	109.5	C20—C19A—H19F	109.5
H12A—C12—H12B	109.5	H19D—C19A—H19F	109.5
C13—C12—H12C	109.5	H19E—C19A—H19F	109.5
H12A—C12—H12C	109.5	C17—C15A—H15D	109.5
H12B—C12—H12C	109.5	C17—C15A—H15E	109.5
C13—C11—H11A	109.5	H15D—C15A—H15E	109.5
C13—C11—H11B	109.5	C17—C15A—H15F	109.5
H11A—C11—H11B	109.5	H15D—C15A—H15F	109.5
C13—C11—H11C	109.5	H15E—C15A—H15F	109.5
H11A—C11—H11C	109.5	C17—C16A—H16D	109.5
H11B—C11—H11C	109.5	C17—C16A—H16E	109.5
C10—C8—H8A	109.5	C17—C16A—H16F	109.5

### Hydrogen-bond geometry (Å, °)

**Table:**

*D*—H···*A*	*D*—H	H···*A*	*D*···*A*	*D*—H···*A*
O2—H2D···S5	0.82	2.47	3.199 (8)	149
O2—H2D···S6	0.82	2.59	3.258 (8)	139
